# Anti-Corrosion Mechanism of Parsley Extract and Synergistic Iodide as Novel Corrosion Inhibitors for Carbon Steel-Q235 in Acidic Medium by Electrochemical, XPS and DFT Methods

**DOI:** 10.3389/fbioe.2021.815953

**Published:** 2021-12-24

**Authors:** Shan Wan, Huikai Chen, Tian Zhang, Bokai Liao, Xingpeng Guo

**Affiliations:** ^1^ Joint Institute of Guangzhou University and Institute of Corrosion Science and Technology, Guangzhou University, Guangzhou, China; ^2^ School of Chemistry and Chemical Engineering, Guangzhou University, Guangzhou, China

**Keywords:** green corrosion inhibitor, electrochemical impedance spectra, potentiodynamic polarization, weight loss method, anti-corrosion mechanism

## Abstract

The parsley extract (PLE) was prepared using absolute ethyl alcohol. The PLE and synergistic iodide were firstly utilized as efficacious corrosion inhibitors to slow down the corrosion rate of carbon steel-Q235 in 0.5 mol/L H_2_SO_4_ solution. The anti-corrosion performance was researched by weight loss method, electrochemical tests, surface analysis and quantum chemistry calculation. Results of electrochemical and weight loss tests show that the synergetic PLE and I^−^ exhibit the optimal corrosion inhibition efficiency 99%. The combined inhibitor displays the favorable long-term corrosion inhibition effect, and the inhibition efficiency can maintain more than 90% after 144 h immersion. The introduction of I^−^ makes carbon steel surface with higher negative charge amount, which could be beneficial to the interaction between corrosion inhibitor and Fe atoms. The adsorption behavior obeys the Langmuir isotherm adsorption, and involves chemical and physical adsorption. On the basis of electrochemical consequences and theoretical calculation, the adsorption process and anti-corrosion mechanisms are further explored.

## 1 Introduction

Corrosion is an inevitable natural issue for most metals according to the laws of thermodynamics, namely conversion from pure metal to their more stable thermodynamic form. However, as for metals used in equipment and buildings, corrosion will bring huge damage to the material service performance. Considering the threat caused by corrosion with economic, material conservation and safety impacts in various engineering applications, the public have developed several proper corrosion prevention methods, including coating, corrosion inhibitor, sacrificial anode, corrosion-resistance alloys, etc. (Nazeer and Madkour, 2018; Wan et al., 2021a; Kang et al., 2021; Salleh et al., 2021; Xu et al., 2021; Cao et al., 2022). Generally, metals are defenceless to corrosion in the presence of acid, which can attack and dissolve metal into ions. Acid solutions have been widely used in industrial occasions, such as acid cleaning, pickling, acidizing, etc. Besides, some acidic contaminants including acetic acid, naphthenic acid, hydrogen sulfide, etc., can easily corrode the metal-based products (Goyal et al., 2018a). One of the practical ways to tackle corrosion for metal under acidic condition is by using corrosion inhibitor due to its low-cost, easy-fabrication and convenient-operation (Wan et al., 2021b; Zeng et al., 2021).

**GRAPHICAL ABSTRACT:**
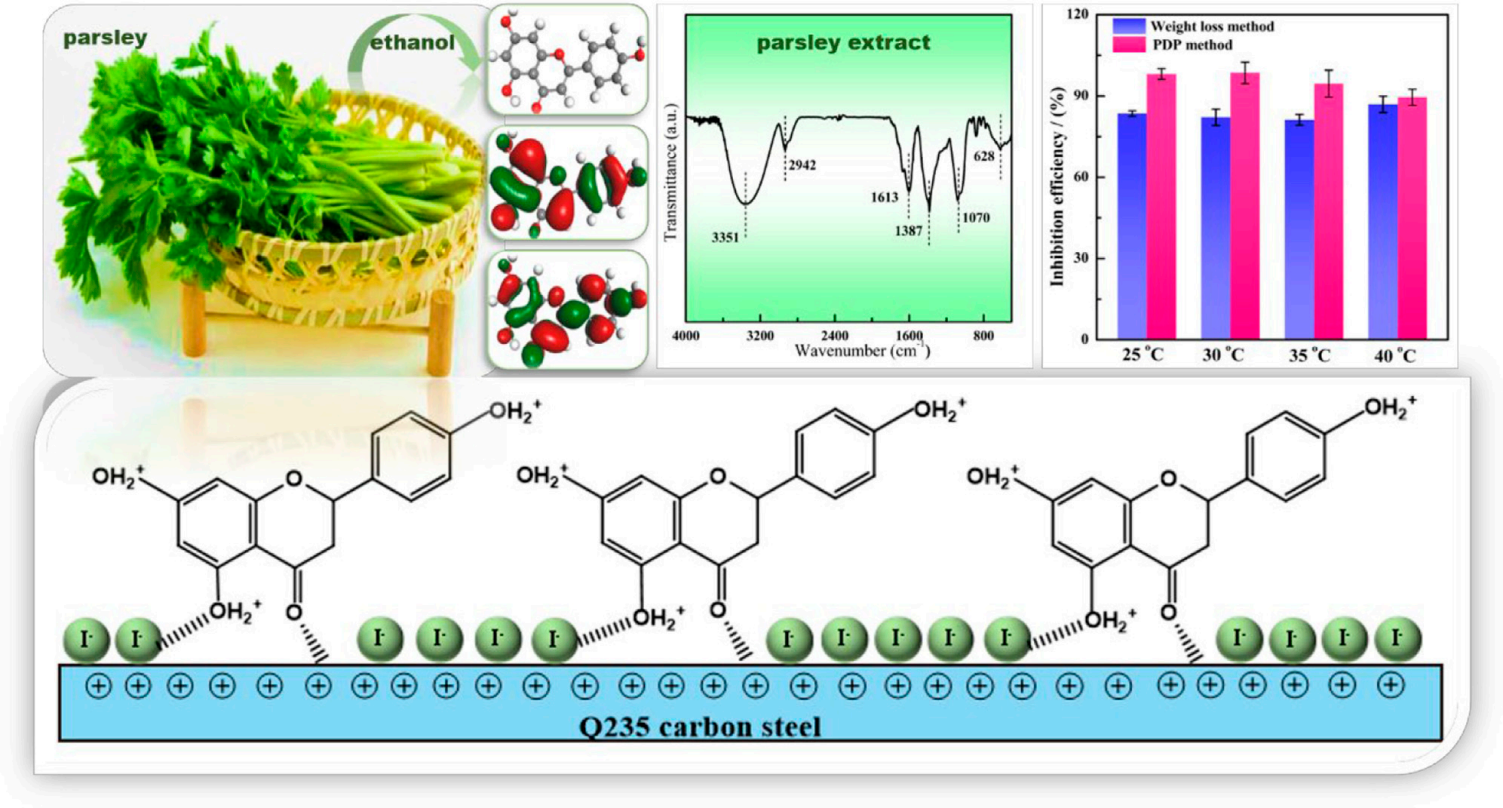


Corrosion inhibitor (Finšgar and Jackson, 2014; Liu et al., 2017; Liu et al., 2019; Obot et al., 2019) can be defined as a substance, which can obviously reduce the corrosion rate of metals with addition of small amount. However, most traditional corrosion inhibitors contain some toxic chemicals, which are harmful to human (cancer, hypertension, etc.) or ecological health (eutrophication, algal bloom, etc.). With the growing awareness of environmental protection, the search for greener alternatives has attracted wild attentions in recent decades. The green corrosion inhibitor (Verma et al., 2018) mainly concerns about nontoxic, eco-friendly processes, renewable source of materials, readily available and low cost. Recently, large numbers of studies have been increasing about utilizing plant extract-based green corrosion inhibitors. Tan et al. (2021a) prepared an aqueous *Brassica oleracea* L. extract and applied it into corrosion prevention of Q235 steel, the best corrosion inhibition efficiency reached 93.8% in HCl and 92.3% in H_2_SO_4_ solutions respectively. Feng et al. (2021) investigated the anticorrosion performance of Veratrum root extract for copper in H_2_SO_4_ solution and the optimum corrosion inhibition efficiency can reach 97% at 200 mg/L. Tan et al. (2021b) extracted papaya leaves using ultra-water and the maximum corrosion inhibition efficiency can achieve 92.5% for copper in 0.5 mol/L H_2_SO_4_ solution.

Parsley belongs to the edible vegetables with ever-increasing usage all year around in the world. It can also act as a medicinal plant with laxative properties and anti-urolithiatic effect (Kreydiyyeh et al., 2001; Al-Yousofy et al., 2017). The parsley extracts contain large amounts of flavonoid compounds with electron-rich aromatic rings and heterogeneous oxygen atoms in their structure, which is in favor of bonding with the empty orbital of metal. Limited studies have reported about corrosion protection performance of the parsley extract (PLE). For example, Benarioua et al. (2019) indicated that the parsley extract was a mixed type inhibitor, and the maximum corrosion inhibition efficiency could reach 92.4% with a 5 g/L concentration in HCl solution at 25°C. Boutoumit et al. (2017) found 1 g/L PLE possessed corrosion inhibition efficiency with 89.5% for carbon steel in HCl solution. Although the PLE was used as a corrosion inhibitor in the past, but the corrosion protection performance was not acceptable, and the relevant corrosion inhibition mechanism is still unclear. The excessive addition amount, unsatisfactory inhibition efficiency and unknown extremely long-term effectiveness restrict the application of PLE as a practical corrosion inhibitor.

In this work, the synergistic influence of PLE and iodide on the corrosion protection of carbon steel-Q235 in H_2_SO_4_ solution was firstly investigated. The inhibition efficiency and long-term effectiveness of PLE were significantly enhanced by the addition of small dose of iodide, which could dramatically lower the dosage of PLE. Weight loss method, electrochemical tests (electrochemical impedance (EIS), potentiodynamic polarization (PDP), Potential of Zero Charge (PZC)), surface characterizations (Fourier Transfer Infrared Spectroscopy (FTIR), Ultraviolet Spectrum (UV), X-ray Photoelectron Spectroscopy (XPS)) and quantum chemical calculations were applied to investigate the structure and composition of PLE, and corrosion inhibition behavior of the new combined inhibitor. The adsorption isotherm and corrosion inhibition mechanism were put forward.

## 2 Experiments

### 2.1 Materials and Reagents

The concentrated sulfuric acid, absolute ethyl alcohol, potassium iodide, and ethyl acetate were purchased by Sinopharm Chemical Reagent Corporation. The electrolytic solution of 0.5 mol/L H_2_SO_4_ was prepared by diluting analytical grade 98% sulfuric acid with deionized water.

The carbon steel-Q235 electrode was sealed in the epoxy resin, leaving a working area of 1 cm × 1 cm Before each test, the working electrodes were abraded by # 400, # 800 and # 1200 SiC sandpapers in sequence. Then the well-polished electrodes are cleaned with absolute ethyl alcohol and acetone for 5 min in turns. Finally, they were dried with cool air and stored in the desiccator.

### 2.2 Parsley Extraction

The fresh parsley was cracked by a knife on the cutting board, and wrapped into a size of filter paper with a diameter of 8 cm. Soxhlet extractor was applied to obtain the PLE taken alcohol as extraction solvent. The acquired solution after 8 h reflux was concentrated, and dissolved in 100 ml deionized water and 100 ml ethyl acetate. Then the mixed solution was transferred into the separating funnel. The aqueous phase was collected and dried to obtain the target product PLE.

### 2.3 Characterizations

The chemical bond of PLE structure was analyzed by FTIR (Spectrum 100, United States) and UV (UV2550, United States) technologies. The composition and micro-morphology of corrosion products on the carbon steel surface in the absence and presence of the PLE inhibitor were characterized by ATR-FTIR, XPS (Escalab250xi, United States of America), SEM (JSM-6701F, Japan) and 3D (VHX-1000E, Keyence, Japan) microscope.

### 2.4 Weight Loss Methods

The carbon steel-Q235 specimens with dimensions of 1 cm × 1 cm × 4 mm were used for weight loss experiments. Firstly, the abraded carbon steel specimens were weighed, and then transferred to the inhibitor-free and inhibitor-containing 0.5 mol/L sulfuric solution under different temperatures after 24 h immersion. Next, the samples were removed from the solution, and the surface corrosion products were cleaned by 1 mol/L HCl solution containing 1 wt% naphthamine. Finally, they were dried by cool nitrogen and re-weighted. To ensure the accuracy of experiment, three parallel samples were used for every test condition. The *w*_*0*_ and *w* are the weight loss of carbon steel before and after 24 h immersion in the sulfuric solution with and without the inhibitor. The corrosion rate (*v*) and inhibition efficiency (*η*_*w*_) are deduced by the following equation:

$$v=\frac{w_{0}-w}{St}\times 100\%$$ (1)

$$\eta_{\text{w}}=\frac{v_{0}-v}{v_{0}}\times 100\%$$ (2)

where *v*_*0*_ and *v* represent the corresponding average corrosion rate, and *S* is the whole area of carbon steel.

### 2.5 Electrochemical Measurements

The electrochemical tests were conducted with a three-electrode system. The carbon steel-Q235 electrode was applied as a working electrode, the counter electrode and reference electrode were Pt conductance electrode and saturated calomel electrode respectively. The EIS test was carried out as a sine wave disturbance of 10 mV versus open circuit potential (OCP) with a wide frequency range from 10,000 to 0.01 Hz. The PDP test was conducted with a polarization scope of ±150 mV vs. OCP at a scanning rate of 0.5 mV/s. All the electrochemical tests were repeated at least three times under the same conditions to ensure the reproducibility.

### 2.6 Quantum Chemical Calculations

The Gaussian 09 software was used to obtain the optimized geometry structure of PLE based on B3LYP/6−311++g (d, p) level of theory, and correlate the PLE structure with corrosion inhibition efficiency and mechanism. Moreover, the molecular characteristic parameters, including the frontier molecule orbitals (FMD), the highest occupied molecular orbital energy (HOMO), the lowest unoccupied molecular orbital energy (LUMO), energy gap (ΔE), dipole moment (μ), electronegativity (Χ), hardness (δ), and charge transfer number (ΔN), were acquired for the analysis of corrosion inhibition performance.

## 3 Results

### 3.1 Fourier Transfer Infrared Spectroscopy and Ultraviolet Spectrum Analysis

**FIGURE 1:**
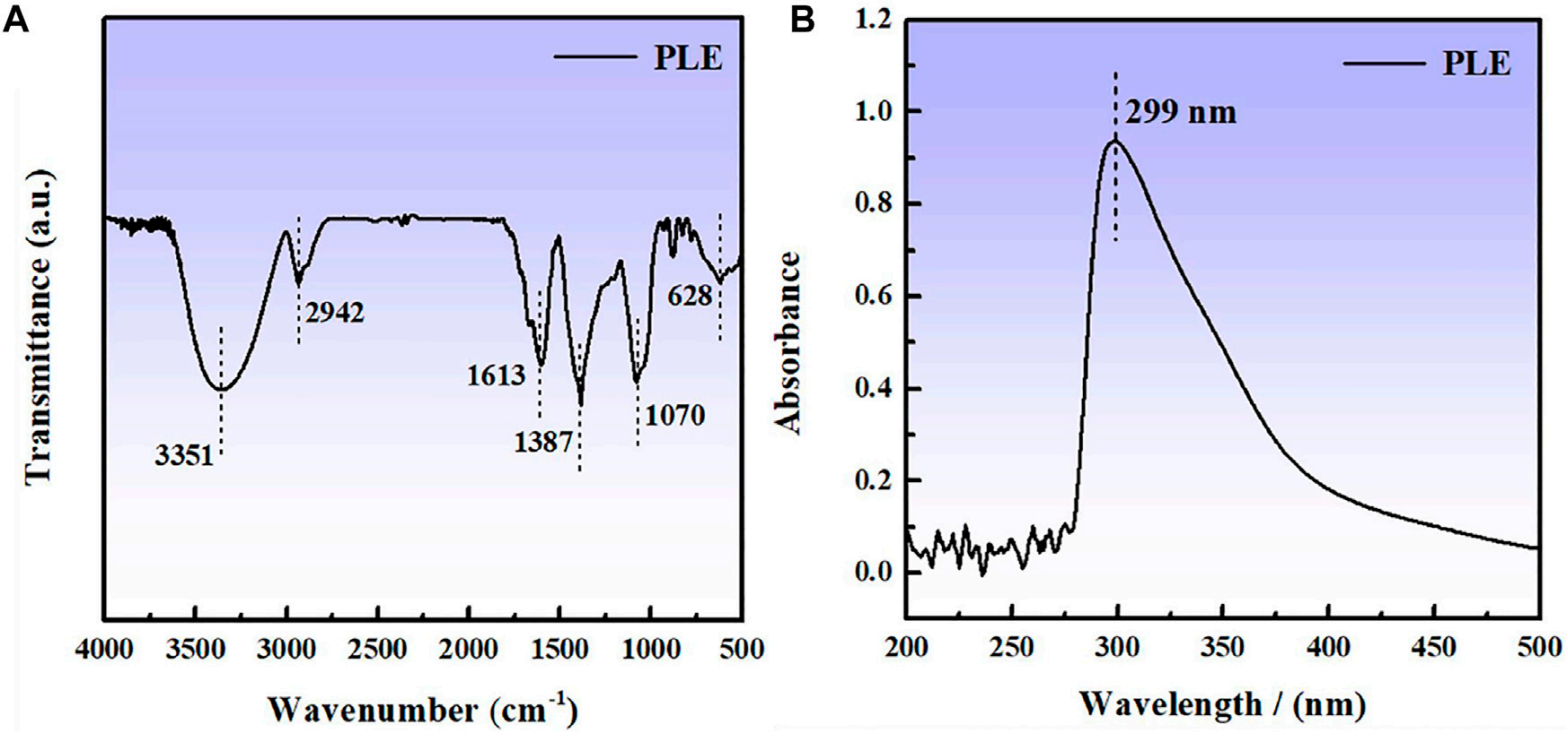
FTIR **(A)** and UV spectrum **(B)** of PLE.

The compositions of PLE were analyzed by the FTIR and UV-vis spectra, as shown in Figures 1A,B. The obvious adsorption peak at 3,351 cm^−1^ is related to the stretching vibration of O-H bond. The appearance of adsorption peaks at 2,942, 1,613, and 1,070 cm^−1^ can be attributed to the stretching vibration of C-H, C=C and C-O bonds, respectively. The peaks located at 1,387 and 628 cm^−1^ could be assigned to the C-H vibration of methyl and on the aliphatic or aromatic groups (Tan et al., 2021b). An obvious peak appears at 299 nm in the UV spectrum in Figure 1B, which corresponds to n-π* transitions (Corrales Luna et al., 2019) of the flavonoid unit in the PLE structure.

**FIGURE 2:**
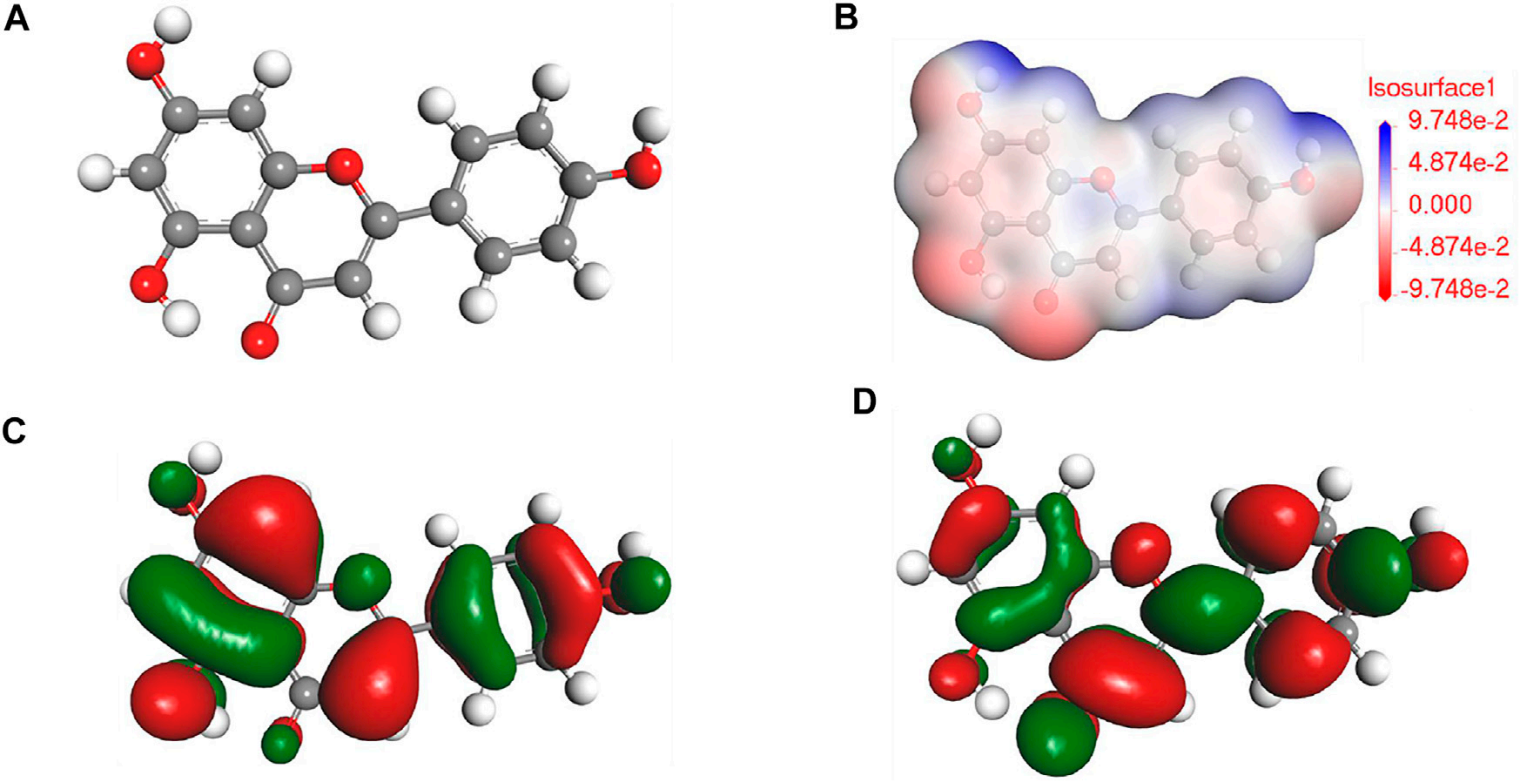
The optimized geometry structure **(A)**, ESP **(B)**, HOMO **(C)**, and LUMO **(D)** of DHBP.

According to the previous reports (Brankovic et al., 2010; Rezazad and Farokhi, 2014) in combination with the results of FTIR and UV results, the main component for PLE can be identified as the 5,7-dihydroxy-2-(4-hydroxyphenyl)-4-benzopyranone (DHBP). Quantum chemical calculation was carried out to research the relation between the structure of DHBP and its corrosion inhibition performance. Figures 2A–D shows the optimized geometry structure, electrostatic potential distribution (ESP) shapes, the highest occupied molecular orbital energy (HOMO), and the lowest unoccupied molecular orbital energy (LUMO) of DHBP. In the ESP shape, the red and green parts represent nucleophilic and electrophilic activities of the DHBP molecule, respectively. The red region is mainly centred on the electronegative oxygen atom, which is in favor of the adsorption on the empty orbital of Fe atom. From Figures 2C,D, the HOMO is primarily distributed on the aromatic ring and carbonyl group and hydroxyl group or oxygen atom in the aromatic ring, while the LUMO is mainly located at surface of O-heterocyclic ring and benzene ring. The corresponding calculated molecular parameters are present in the Table 1. The E_HOMO_ and E_LUMO_ values represent the ability of electron-donating and electron-accepting. Generally, the lower value of energy gap signifies easier adsorption on the metal surface and higher protection effectiveness (Luo et al., 2021). Owing to that dense electron-rich regions appear around O atoms from the HOMO orbital of DHBP, it could be concluded that DHBP can adsorb on the surface of carbon steel through formation of covalent bonds between lone pairs from oxygen atoms and unoccupied orbitals from Fe atoms. Moreover, the dipole moment (*μ*) value is an indicator to evaluate the intermolecular interactions and the forces of dipole-dipole. Some reports have pointed out that a lower *μ* value means stronger reaction activity and is beneficial to adsorption on the surface of carbon steel (Musa et al., 2012; Majd et al., 2019).

**TABLE 1:** Molecular properties of PLE extract calculated using DFT.

PLE	*E*_*HOMO*_ (eV)	*E*_*LUMO*_ (eV)	*ΔE* (eV)	μ (debye)
DHBP	−6.228	−2.039	4.189	7.69

### 3.2 Electrochemical Analysis

**FIGURE 3:**
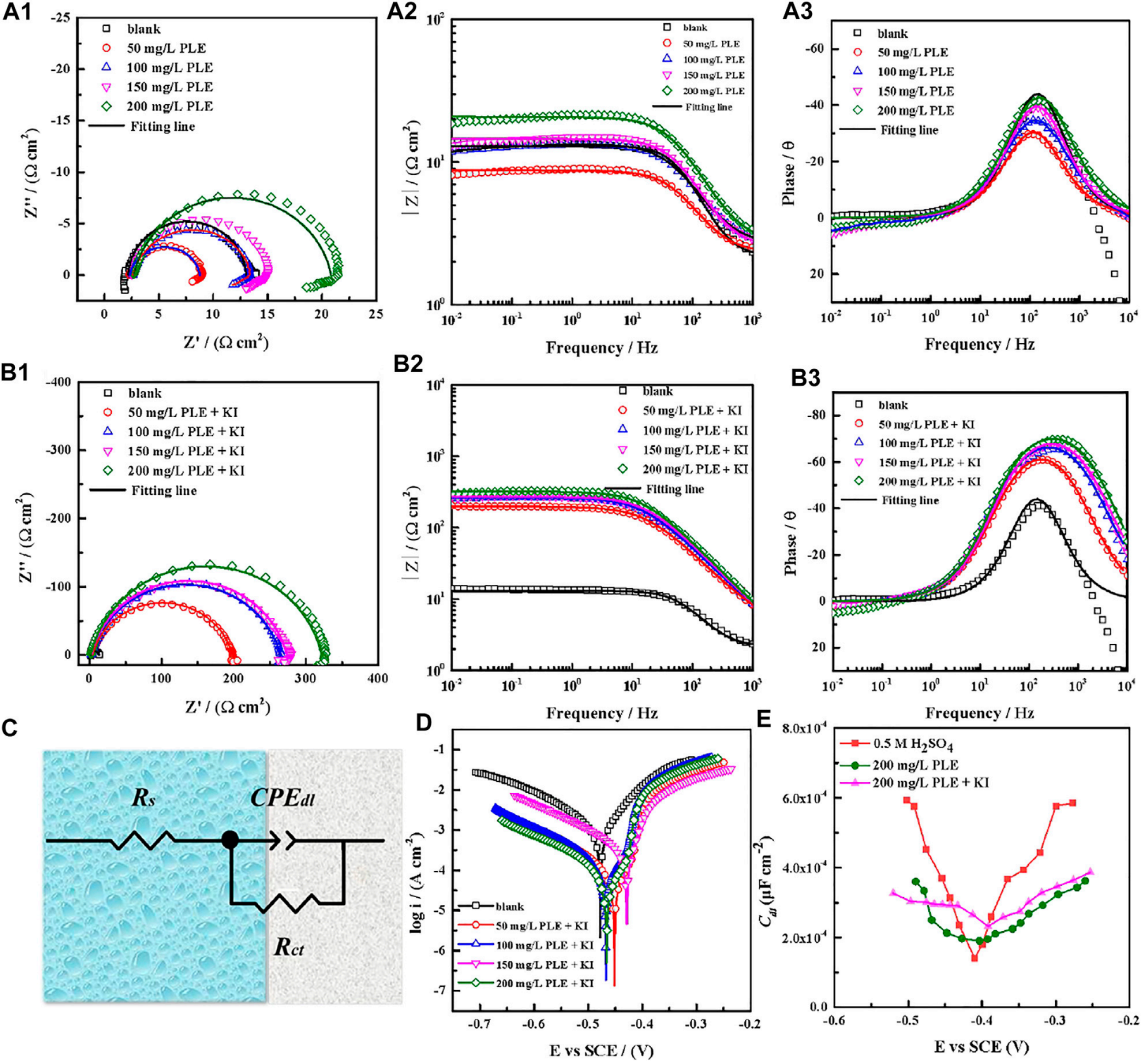
EIS plots of carbon steel-Q235 in 0.5 mol/l H_2_SO_4_ solution containing PLE **(A1–A3)**, PLE + KI **(B1–B3)**, corresponding equivalent electrical circuit **(C)**, polarization curves **(D)**, and differential capacitance curves **(E)**.

The corrosion behaviour of carbon steel-Q235 in 0.5 mol/L H_2_SO_4_ solution was investigated by EIS, PDP and differential capacitance curves methods under different concentrations of PLE and PLE + KI. The EIS plots, corresponding equivalent electrical circuit, polarization curves and differential capacitance curves are shown in Figure 3. From Figures 3A1–A3, the diameter of capacitive reactance in the EIS plots only increases slightly with an increasing concentration of PLE. After the introduction of KI, the impedance value at 0.01 Hz significantly augments by one or two orders of magnitude. All EIS diagrams only have a capacitive reactance arc, which can correspond to charge transfer resistance (*R*_*ct*_) and double electrical layer capacitance (*CPE*_*dl*_). The corresponding fitted parameters are present in Table 2. The inhibition efficiency (*η*) of PLE and PLE + KI could be defined as:

$$\eta =\frac{R_{ct}-R_{ct}^{0}}{R_{ct}}\times 100\%$$ (3)

where *R*_*ct*_ and $R_{ct}^{0}$ represents the charge transfer resistance with and without corrosion inhibitor. In individual presence of PLE, inhibition efficiency only reaches 42% at 200 mg/L concentration, while under the synergistic effect of PLE and KI, the inhibition efficiency markedly increases to 97%, suggesting that iodide can accelerate the formation of protective film of PLE on the carbon steel surface.

**TABLE 2:** The corresponding EIS fitting results in Figure 3.

System	*R*_*s*_ (Ω cm^2^)	*CPE*_*ct*_ *-T* (S^n^ Ω^−1^ cm^−2^)	*CPE*_*ct*_ *−n*	*R*_*ct*_ (Ω cm^2^)	*η* (%)
0.5 M H_2_SO_4_	2.16	0.00034	0.88	11	—
50 mg/L PLE	2.31	0.00095	0.87	7	—
100 mg/L PLE	2.62	0.00063	0.86	11	—
150 mg/L PLE	2.55	0.00046	0.90	13	15
200 mg/L PLE	2.68	0.00040	0.86	19	42
50 mg/L PLE + KI	4.05	9.32 × 10^–5^	0.85	194	94
100 mg/L PLE + KI	2.37	7.68 × 10^–5^	0.85	262	96
150 mg/L PLE + KI	2.21	6.45 × 10^–5^	0.86	271	96
200 mg/L PLE + KI	1.86	5.72 × 10^–5^	0.87	320	97

The results of PDP tests were shown in Figure 3D. Obviously, the corrosion potential shifts slightly after addition of PLE + KI, exhibiting the property of mixed-type inhibitor. Anodic and cathodic processes are both inhibited in the presence of PLE + KI, according to the significant decrease of both anodic and cathodic corrosion current density. Based on the Tafel extrapolation method, electrochemical parameters of PDP curves are summarized in Table 3. The corresponding inhibition efficiency (*η*_*p*_) of the inhibitors could be interpreted into:

$$\eta_{p}=\frac{i_{corr}^{0}-i_{corr}}{i_{corr}^{0}}\times 100\%$$ (4)

where $i_{corr}^{0}$ and *i*_*corr*_ represent the corrosion current density in the absence and presence of the inhibitor. From Table 3, *i*_*corr*_ decreases from 1.6 × 10^−3^ A/cm^2^ to 5.59 × 10^−5^ A/cm^2^ after adding 200 mg/L PLE + KI in sulfuric solution. The corresponding *η*_*p*_ value reaches 99.7%, suggesting the outstanding inhibition performance of PLE + KI.

**TABLE 3:** The corresponding PDP fitting results in Figure 3.

System	*B*_*a*_ (mV/dec)	*B*_*c*_ (mV/dec)	*I*_*o*_ (A/cm^2^)	*E*_*o*_ (Volts)	*η*_*p*_ (%)
0.5 M H_2_SO_4_	94	168	0.0016	−0.48	—
50 mg/L PLE + KI	82	212	0.00055	−0.43	96.5
100 mg/L PLE + KI	53	121	7.17 × 10^–5^	−0.45	99.6
150 mg/L PLE + KI	60	115	8.85 × 10^–5^	−0.47	99.4
200 mg/L PLE + KI	64	132	5.59 × 10^–5^	−0.47	99.7

The electrochemical impedance tests at various bias voltages (−300–300 mV) in the presence and absence of PLE and PLE + KI were carried out to obtain differential capacitance curves and PZC. It is seen from Figure 3E that in the absence and presence of PLE and PLE + KI, the PZC values are −0.4831, −0.4036, and −0.4457 V, while the OCP values show −0.5019, −0.4682, and −0.4537 V, respectively. The Antropov’s rational corrosion potential (Ψ) is deduced by the difference value between the OCP and PZC (Chidiebere et al., 2014). After the introduction of PLE or PLE + KI, the carbon steel surface carries the positive charge in comparison with the negative charge in blank solution. And the addition of I^−^ results in a lower Ψ value and the higher negative charge amount, suggesting the more favorable adsorption for the protonated PLE inhibitor on the carbon steel surface.

**FIGURE 4:**
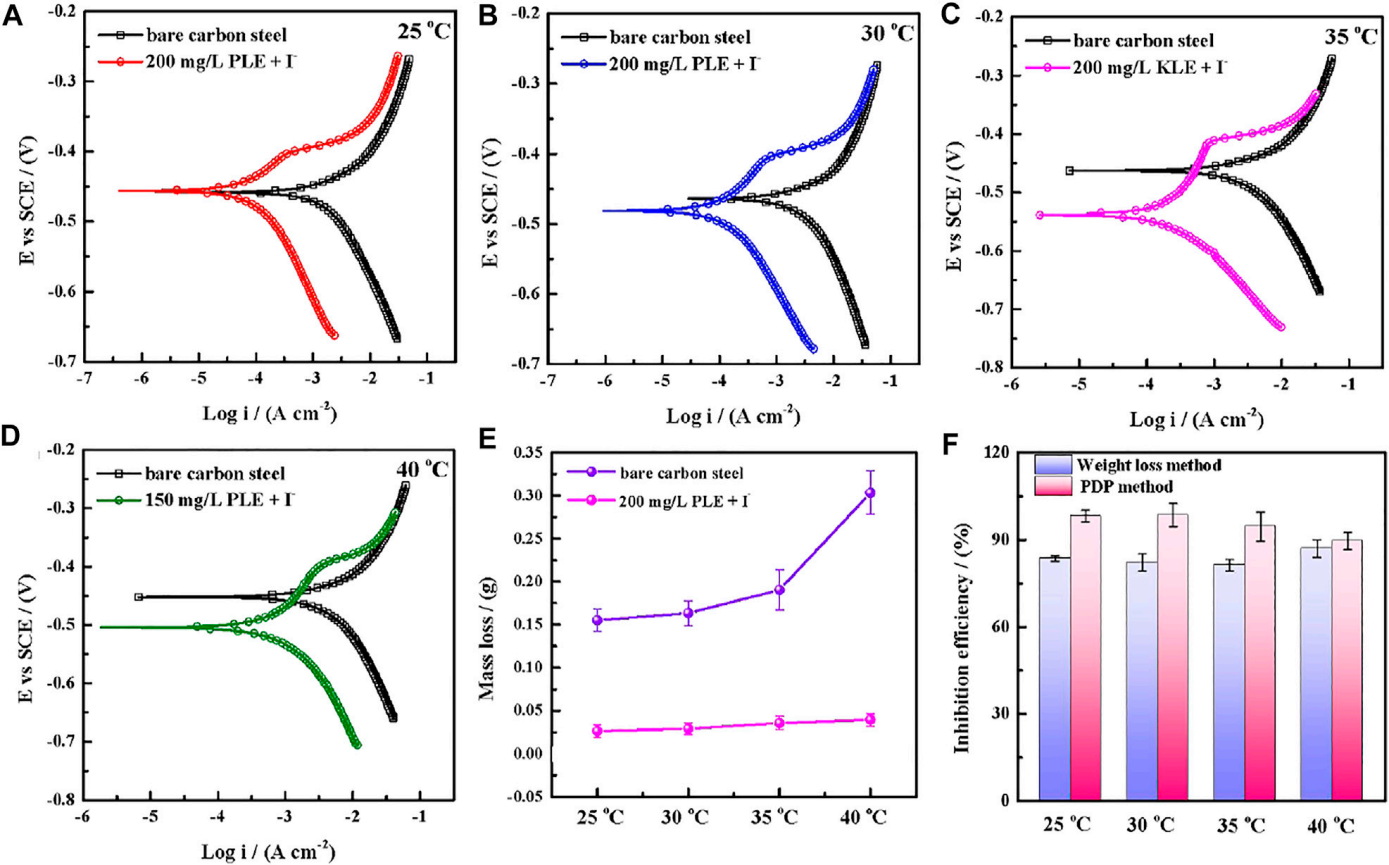
Potentiodynamic polarization curves for carbon steel-Q235 after 24 h immersion in 0.5 mol/L H_2_SO_4_ solution in the absence and presence of 200 mg/L PLE +60 mg/L KI under different temperature: 25°C **(A)**, 30°C **(B)**, 35°C **(C)**, 45°C **(D)**; corresponding weight loss curves **(E)** and comparative curves of inhibition efficiency **(F)** by weight loss method and PDP method.

The PDP and weight loss methods are adopted to research corrosion behaviors of carbon steel in the absence and presence of 200 mg/L PLE +60 mg/L KI under different temperature, as shown in Figure 4. From Figures 4A–D, after the introduction of PLE and KI, the corrosion current density significantly decreases. With temperature rising, corrosion potential subsequently shifts to the negative direction, and corrosion current density slightly augments. Moreover, the amplitude of the potential movement is less than 85 mV (Lima et al., 2020), implying that it belongs to the mixed-type inhibitor (Hsissou et al., 2020). Figure 4E presents the weight loss results after 24 h immersion. The weight loss decreases generally with the rising temperature. Specially, when temperature increases from 35 to 40°C, the weight loss dramatically augments in blank sulfuric solution, mainly attributing to magnification of anodic and cathodic reaction rate constant. Figure 4F summarizes the consequences of inhibition efficiency by PDP and weight loss methods. The detailed weight loss results are presented in Table 4. The obtained inhibition efficiency values fluctuate around 90%, suggesting excellent corrosion protection performance. The inhibition efficiency results by PDP method are a little more than those by weight loss method. Their differences are ascribed to that the former is instantaneous, while the latter is average.

**TABLE 4:** The weight loss of carbon steel-Q235 in the uninhibited and inhibited solutions under different temperature.

Temperature/(°C)	Weight loss/(g)	
	Uninhibited solution	Inhibited solution
25	0.1551	0.02630
30	0.1635	0.02925
35	0.1905	0.03570
40	0.3031	0.03955

**FIGURE 5:**
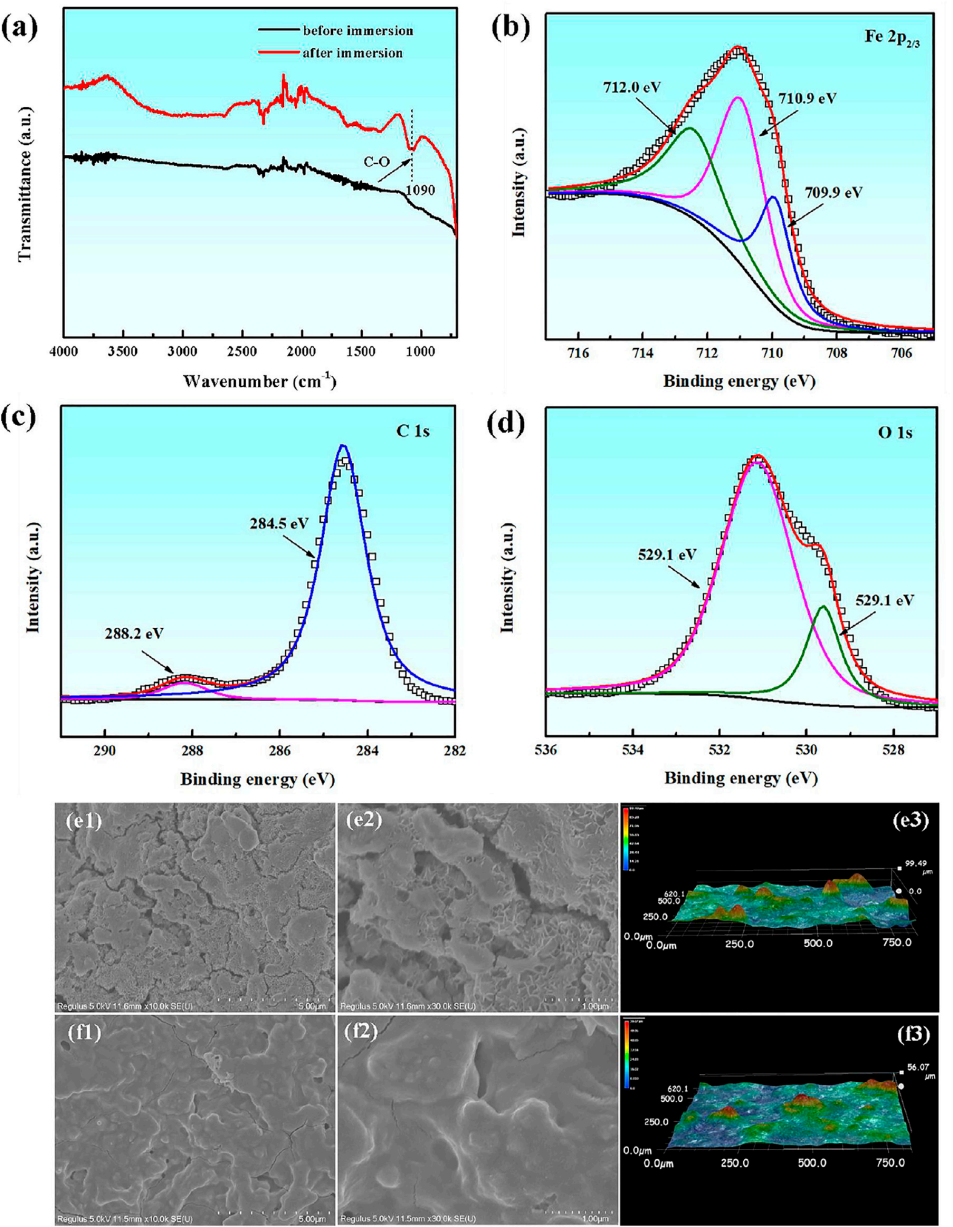
ATR-FTIR spectra **(A)**, XPS spectra of Fe 2p **(B)**, C 1s **(C)** and O 1s **(D)** element of surface corrosion products for carbon steel after 24 h immersion in 0.5 mol/L H_2_SO_4_ solution containing 200 mg/L PLE +60 mg/L KI; corresponding SEM and 3D morphologies in the absence **(E1–E3)** and presence **(F1–F3)** of the inhibitor.

The compositions and micro-morphologies of corrosion products on the carbon steel surface are analyzed by the ATR-FTIR spectra, UV spectra and SEM microscope after 24 h immersion in the presence of PLE and KI. From Figure 5A, it is obvious that the adsorption peak at 1,090 cm^−1^ appears, which is related to the stretching vibration of C-O in the PLE structure. Figures 5B–D shows corresponding XPS spectra of Fe 2p, C 1s and O 1s of surface corrosion products. From Fe 2p_3/2_ spectrum, it shows three deconvoluted peaks at 709.9, 710.9 and 712.0 eV (Zhang et al., 2021a), which could be ascribed to FeCO_3_, Fe_2_O_3_ and FeOOH, respectively. The C 1s spectrum can be deconvoluted into two peaks at 288.2 and 284.5 eV, which could be assigned to FeCO_3_ and C-C/C-H (Zhang et al., 2021b), confirming the adsorption of PLE on the carbon steel surface. Similarly, there are also two peaks appearing in the O 1s spectrum, corresponding to FeO/Fe_2_O_3_ at 529.1 eV and C=O at 531.1 eV, respectively (Chidiebere et al., 2014). SEM analysis was performed to further research the differences in micro-morphologies with and without of PLE and I^−^, as shown in Figures 5E,F. In the absence of inhibitors, corrosion products are loose with lots of cracks, which presents laminar microstructure with a roughness of 99.49 μm. After adding the inhibitor, corrosion products become dense with tiny bumps. The corrosion product film exhibits a more smooth topography compared with that in the uninhibited solution, and corresponding surface roughness decreases to 56.07 μm. The SEM surface micromorphology further verifies that PLE and I^−^ retard the corrosion of carbon steel effectively.

### 3.3 Long-Term Corrosion Resistance

**FIGURE 6:**
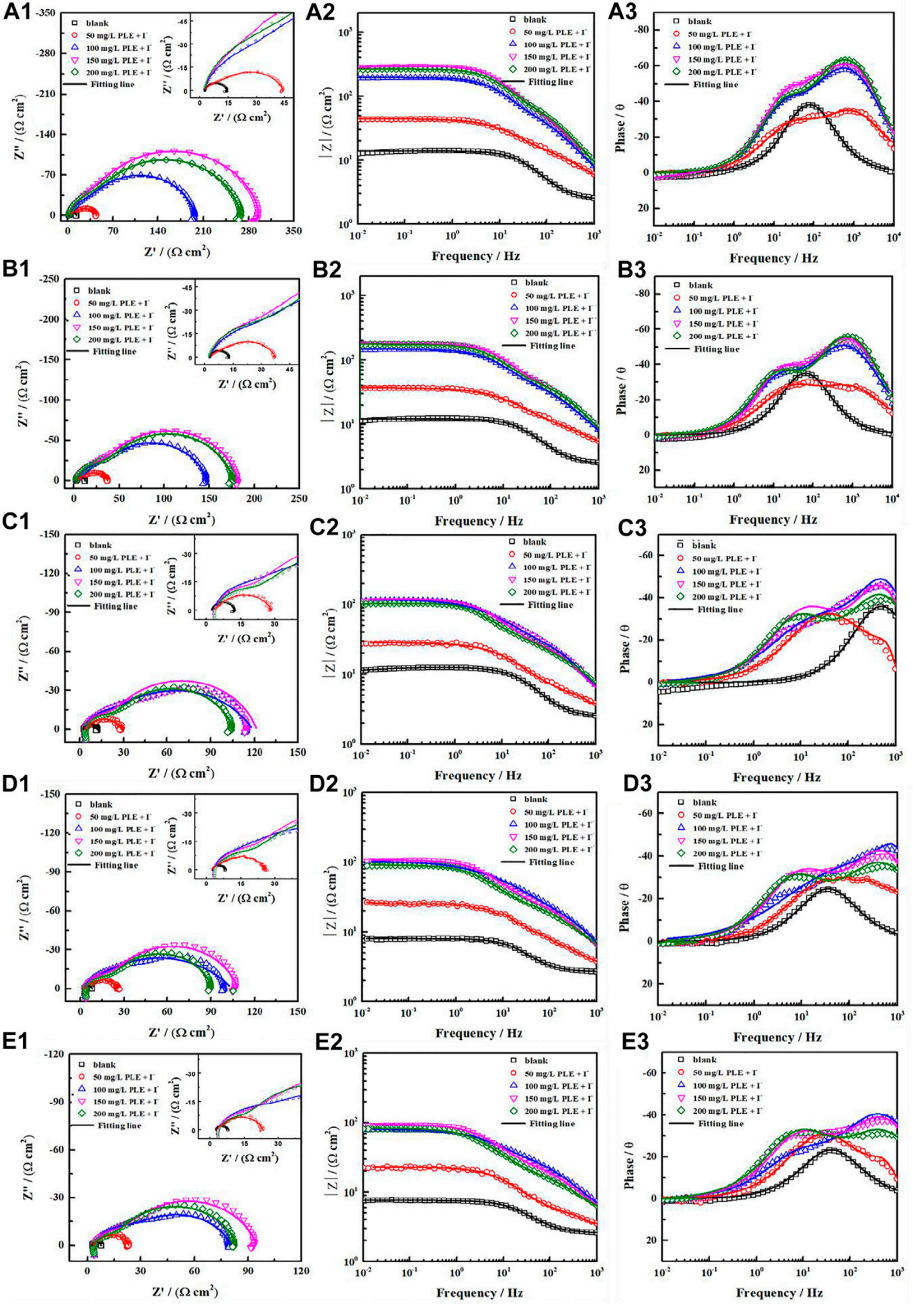
The variations of Nyquist and Bode plots versus time for carbon steel without and with PLE and KI inhibitors under different immersion time in 0.5 mol/L H_2_SO_4_ solution: 24 h **(A1–A3)**, 48 h **(B1–B3)**, 96 h **(C1–C3),** 120 h **(D1–D3)**, 144 h **(E1–E3)**.

**FIGURE 7:**
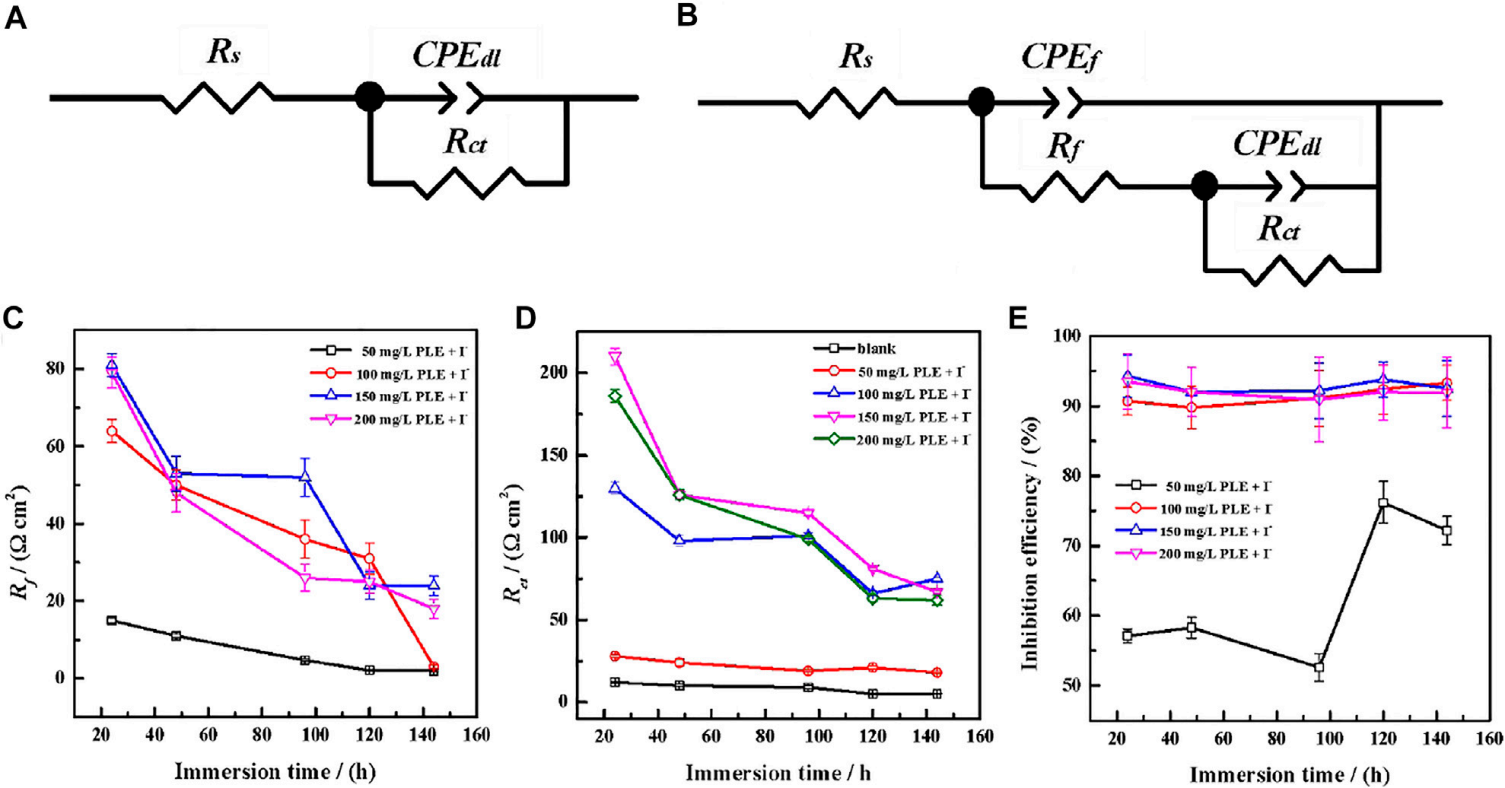
The equivalent circuits of EIS plots in the absence **(A)** and presence **(B)** of the inhibitors after long-term immersion in the sulfuric solution; corresponding variations of Rf **(C)**, Rct **(D)** and inhibition efficiency **(E)** versus immersion time.

Generally, the persistence of inhibition efficiency for green corrosion inhibitors is not desirable, mainly attributing to the instability of corrosion inhibitors and their adsorption film (Chaubey et al., 2021; Salleh et al., 2021). To evaluate the long-term corrosion resistance of PLE and KI, EIS was carried out to *in situ* monitor their inhibition efficiency on carbon steel under different immersion time shown in Figure 6. In uninhibited solution, all EIS plots show one capacitive reactance, corresponding to charge transfer resistance and double electrical layer capacitance. It can be fitted by the equivalent circuit in Figure 7A. After adding the inhibitor, it is obvious that two time constants appear in all the EIS diagrams. The capacitive arcs at high and low frequency could be attributed to the adsorption film and double electrical layer respectively, fitted by equivalent circuit in Figure 7B. To further analyze the results of EIS diagrams under different immersion time, the *R*_*ct*_, *R*_*f*_ (film resistance) and *η* are extracted in Figures 7C–E. With the extend of immersion time, all impedance values decrease under different concentrations of PLE and KI. When adding 50 mg/L PLE + I^−^, the film resistance *R*_*f*_ is 18 Ω cm^2^ after 24 h immersion and decreases with immersion time prolonging, while *R*_*ct*_ value basically keeps invariable. With an increase of the inhibitor concentration, variation tendencies of *R*_*ct*_ and *R*_*f*_ are consistent, namely all obvious decrease with immersion time prolonging. The corresponding *η* results under different immersion time and concentrations are exhibited in Figure 7E. At 50 mg/L PLE + I^−^, *η* firstly decreases in preliminary stage and then slightly increases in the later immersion stage. While the inhibitor concentration significantly increases to 100 mg/L PLE + I^−^, *η* maintains more than 90% during the whole 140 h immersion, suggesting the persistence of corrosion protection performance.

In comparison with inhibition efficiency results from EIS and PDP tests from some previous studies, the inhibition efficiency of PLE + I^−^ inhibitors was relatively higher under similar conditions (Table 5).

**TABLE 5:** Summary of the inhibition efficiency compared with some green corrosion inhibitors.

Inhibitor	Concentration (mg/L)	Metal/Medium	*η*_*EIS*_(%)	*η*_*Tafel*_ (%)	Immersion time (h)	References
Licorice plant extract	1,000	Steel/chloride-polluted concrete pore solution	81	74	24	Naderi et al. (2021)
Rhoeo discolor plant leaves	2,000	Mild steel/0.5 M HCl	87	88	24	Gayakwad et al. (2021)
Chamomile flower	600	Mild steel/HCl	98	98	8	Shahini et al. (2021)
*Syzygium cumini* leaf	300	Carbon steel/acidic medium	72	84	24	Silva et al. (2021)
Jackfruit pectin	1,000	Mild steel/0.5 M HCl	90	90	24	Shamsheera et al. (2022)
*Hymenaea stigonocarpa* Fruit shell	1,233	Steel/sulfuric solution	79	87	12	Policarpi and Spinelli (2020)
PLE + KI	200	carbon steel/sulfuric acid	97	99.7	24	—

### 3.4 Adsorption Isotherm and Corrosion Kinetics

Generally, the adsorption of corrosion inhibitor contains physical and chemical adsorption (Tan et al., 2021b). The electrostatic interaction between charged corrosion inhibitor and metal surface belongs to physisorption, while the formation of coordination bonds between lone pair electrons from heteroatoms in the organic inhibitor and unoccupied orbitals on the metal surface is ascribed to chemical adsorption (Goyal et al., 2018b; Wan et al., 2021c). The premise of adsorption process for corrosion inhibitor is that the inhibitor can replace water molecules and adsorb on the metal surface. To further research the adsorption mechanism of corrosion inhibitor, various models of adsorption isotherm are adopted to fit the surface coverage from polarization curve data with concentration of the inhibitor, which could be illustrated as follows (Tan et al., 2019):

Langmuir model:

$$\frac{C}{\theta }=\frac{1}{K_{\mathrm{ads}}}+C$$ (5)

Temkin model:

$${\text{e}}^{-2\alpha \theta }=KC$$ (6)

Frumkin model:

$$\ln\left[\frac{\theta }{\left(1-\theta \right)C}\right]=\ln K+2\alpha \theta$$ (7)

**FIGURE 8:**
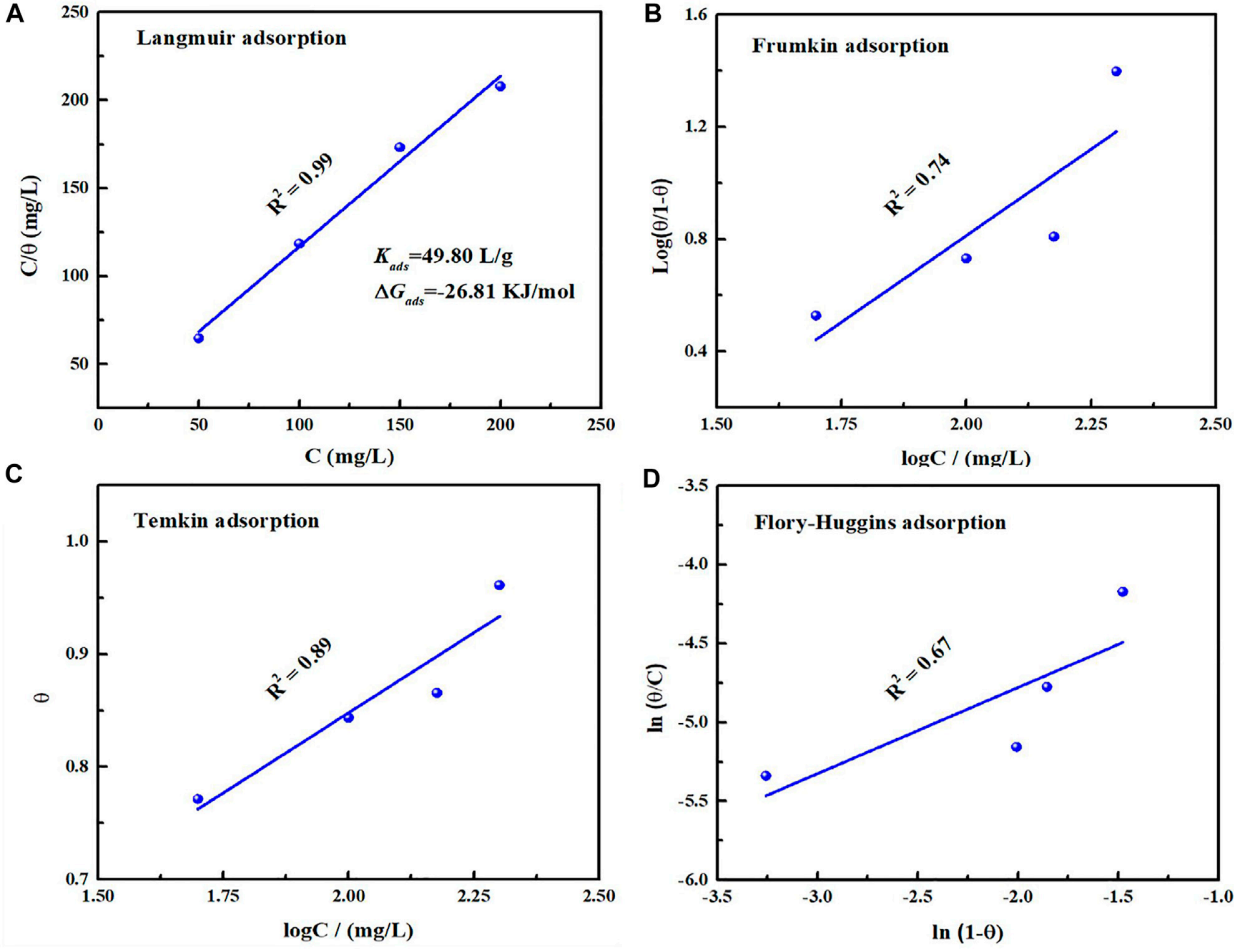
Fitted results based on Tafel polarization curves with different adsorption models: Langmuir **(A)**, Frumkin **(B)**, Temkin **(C)**, Flory-Huggins **(D)** for carbon steel in 0.5 mol/L H_2_SO_4_ solution containing 200 mg/L PLE + I^−^.

Flory-Huggins model:

$$\mathrm{In}\frac{\theta }{C}=x\mathrm{In}\left(\mathrm{1-}\theta \right)+\mathrm{In}\left(xK_{\mathrm{ads}}\right)$$ (8)

where *θ* and K_ads_ represent surface coverage and equilibrium constant of adsorption, and *C* is concentration of corrosion inhibitor. The corresponding fitting results from Langmuir, Frumkin, Temkin and Flory-Huggins adsorption isotherm models are present in Figure 8. Obviously, Langmuir adsorption isotherm is the most suitable and its linear regression coefficient *R* ^2^ is close to 1. According to Eq. 5, K_ads_ value can be deduced as 49.80 L/g. To be aware of adsorption type on the metal/solution, the standard Gibbs free energy of adsorption (∆G_ads_) could be evaluated by the following equation (Zhang et al., 2018):

$$\Delta G_{ads}=-RT\ln\left(55.5K_{ads}\right)$$ (9)

where *R* and *T* represent universal gas constant and thermodynamic temperature respectively. The calculated ∆*G*_*ads*_ value is −26.81 KJ/mol, and the negative value suggests that the inhibitor could adsorb on the metal surface spontaneously. Moreover, in view of that the ∆*G*_*ads*_ value is between −20 and −40 KJ/mol (Li et al., 2018), it involves chemical and physical adsorption on the metal/solution interface.

**FIGURE 9:**
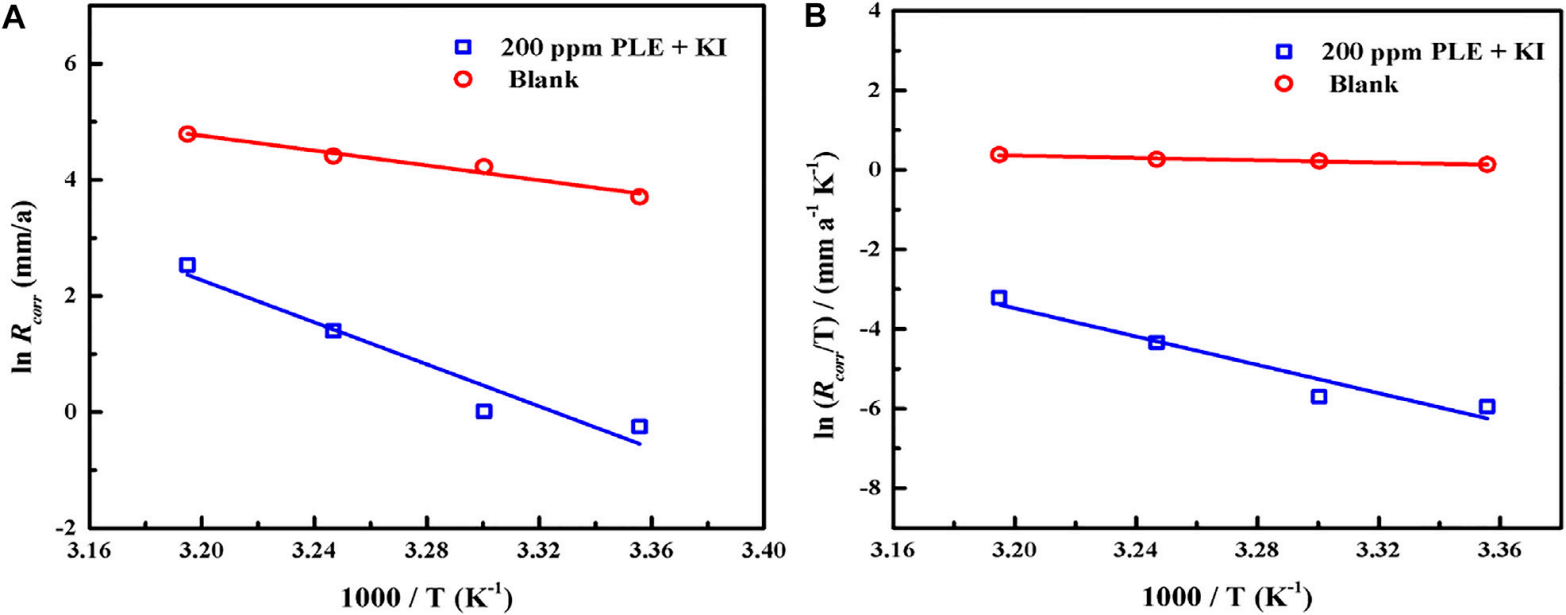
The linear fitting of Arrhenius equation **(A)** and transition state equation **(B)** for carbon steel in 0.5 mol/L H_2_SO_4_ solution containing 200 mg/L PLE + I^−^.

According to PDP results under different temperatures, the adsorption and inhibition performance of corrosion inhibitor was significantly influenced. To make clear the corrosion kinetic process, the adsorption activation energy *E*_*a*_, the enthalpy of activation *∆H*_*a*_, and the entropy of activation *∆S*_*a*_ were put forward according to Arrhenius equation and the Erying transition state equation (Guerraf et al., 2018):

$$\mathrm{InCR}=\mathrm{InA}-\frac{E_{a}}{RT}$$ (10)

$$\mathrm{CR}=\frac{\mathrm{RT}}{\mathrm{Nh}}\exp\frac{\Delta S_{a}}{R}\exp\frac{-\Delta H_{a}}{RT}$$ (11)

where CR stands for the corrosion rate of carbon steel, and *A* represents the pre-exponential factor. The *N*, *h*, *T* and *R* are Avogadro number, Planck’s constant, thermodynamic temperature and universal gas constant, respectively. Figure 9 shows the linear fitting plots of Arrhenius equation and transition state equation for carbon steel in 0.5 mol/L H_2_SO_4_ solution containing 200 mg/L PLE + I^−^. The corresponding linear fitting results are present in Table 6. Generally, *E*_*a*_ could be used to evaluate the difficulty of corrosion reaction. The *E*_*a*_ value (150.52 KJ/mol) in the inhibited solution is higher than that (53.52 KJ/mol) in blank solution, suggesting that the introduction of the inhibitors obviously impedes the corrosion process. The both positive *∆H*_*a*_ values with and without inhibitors indicate that the dissolution of carbon steel possesses an endothermic property. Thus, it could be speculated that elevated temperature is beneficial to the metal dissolution and corrosion process, which is in accordance with the weight loss and PDP results. The negative *∆S*_*a*_ value in uninhibited solution indicates that the corrosion of carbon steel is a process with the reduction of disorder degree. While, *∆S*_*a*_ value in inhibited solution becomes positive, implying that it is an entropy increase process. It could mainly be attributed to the competitive adsorption and desorption of the inhibitor on the carbon steel surface.

**TABLE 6:** Corrosion kinetic parameters of carbon steel with and without the 200 mg/L PLE + I^−^ inhibitors in Figure 9.

System	E_a_/(KJ/mol)	ΔH_a_/(KJ/mol)	ΔS_a_/(KJ^−1^/mol^−1^)
0.5 M H_2_SO_4_	53.25	41.92	−155.64
200 m/L PLE + I	150.52	147.98	247.42

## 4 Discussion

**FIGURE 10:**
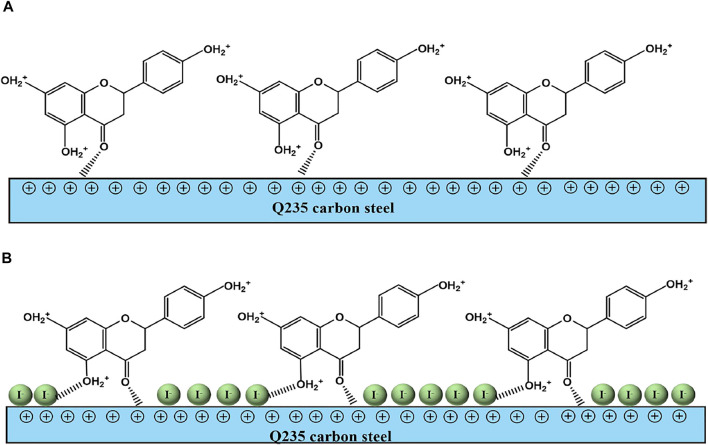
The schematic diagram of synergistic inhibition mechanism of PLE and KI on the surface of carbon steel-Q235 in 0.5 mol/L H_2_SO_4_ solution.

Based on all the electrochemical tests and theoretical calculation results, a possible anticorrosion mechanism is put forward to explain the synergistic effect of PLE and I^−^, as shown in Figure 10. In the blank sulfuric solution, carbon steel surface is susceptible to severe corrosion because corrosion media (H^+^, SO_4_^2−^, H_2_O) can get access to the surface of carbon steel easily (Aadad et al., 2021; Abd El-Lateef and Khalaf, 2021). After adding PLE inhibitor, corrosion process is slightly impeded, while the inhibition effects dramatically enhanced under the coexistence of PLE and I^−^ inhibitors, according to the results of EIS, PDP and weight loss consequences.

On the basis of DFT calculation in Figure 2, there are high electron cloud density distributions around carbonyl group and hydroxyl group or oxygen atom in the aromatic ring of DHBP molecules, which could offer lone pair electrons to bond with the unoccupied orbits of Fe atoms. The formation of PLE adsorption film hinders the intrusion of corrosive ions on the carbon steel surface. Besides, in the inhibited solution, DHBP molecules can replace water molecules and adsorb on the surface of carbon steel as follows (Haldhar et al., 2018):

$${\mathrm{Inh}}_{sol}+{\left[Fe-H_{2}O\right]}_{ads}⇌{\left[Fe-Inh\right]}_{ads}+H_{2}O$$ (12)

From EIS results in Figure 3, individual addition of PLE only improves corrosion protection performance slightly. Because electrostatic repulsion brings about that protonated DHBP molecules in acidic medium are difficult to bond on the carbon steel surface with positive charge (Figure 3E). With further introduction of I^−^, it could replace water molecules and preferentially adsorb on the carbon steel surface due to its larger ionic radius, high hydrophobicity, and low electronegativity compared to SO_4_^2−^ (Solomon et al., 2018). According to differential capacitance curves, absorbed I^−^ ions make carbon steel surface with higher negative charge amount in comparison with that in individual PLE solution. It will contribute to PLE adsorption on the carbon steel surface firmly. According to the fitting results of adsorption isotherm (Figure 8), their adsorption process involves chemisorption and physisorption (Chai et al., 2019). XPS results confirmed the bonding between lone pairs from protonated PLE molecules and empty orbits from Fe atoms, which could be enhanced by absorbed I^−^. Therefore, PLE and synergistic I^−^ dramatically improve corrosion inhibition performance on the surface of carbon steel.

## 5 Conclusion

In this work, PLE was prepared through an alcohol extraction method, and used as an effective corrosion inhibitor with I^−^ for enhancing anti-corrosion performance of carbon steel-Q235 in 0.5 mol/L H_2_SO_4_ solution. The electrochemical tests show that individual PLE only improve corrosion protection performance slightly, while it with I^−^ significantly enhances the inhibition efficiency for carbon steel, and the optimal inhibition efficiency reaches about 99%. Moreover, PLE and I^−^ still maintain high inhibition efficiency 90% after 144 h long-term immersion. The adsorption process obeys the Langmuir isotherm adsorption, and involves chemical and physical adsorption. From the differential capacitance curves, the introduction of I^−^ makes carbon steel surface with higher negative charge amount, which is conducive to the interaction between protonated PLE and Fe atom, namely the π-π conjugation in the benzene ring and σ-π hyper-conjugation between the benzene ring and carbonyl, epoxy or phenolic hydroxyl groups. In addition, the absorbed I^−^ ions on the carbon steel surface make for the adsorption of protonated PLE through electrostatic attraction. The two synergistic effects of PLE and I^−^ endow carbon steel excellent protection performance.

## Data Availability

The original contributions presented in the study are included in the article/Supplementary Material, further inquiries can be directed to the corresponding authors.
